# AAV9-mediated functional screening for cardioprotective cytokines in Coxsackievirus-B3-induced myocarditis

**DOI:** 10.1038/s41598-022-11131-w

**Published:** 2022-05-04

**Authors:** Paolo Carai, Giulia Ruozi, Alexandra Paye, Yannick Debing, Francesca Bortolotti, Julie Lecomte, Lorena Zentilin, Elizabeth A. V. Jones, Mauro Giacca, Stephane Heymans

**Affiliations:** 1 Department of Cardiovascular Sciences, Center for Vascular and Molecular Biology, KU Leuven, Leuven, Belgium; 2 CARIM, Maastricht University, Maastricht, The Netherlands; 3 International Centre for Genetic Engineering and Biotechnology (ICGEB), Trieste, Italy; 4 King’s College London, British Heart Foundation Centre of Research Excellence, School of Cardiovascular Medicine and Sciences, London, UK; 5 Center for Heart Failure Research, CARIM, Department of Cardiology, Maastricht University Medical Center, PO BOX 5800, 6202 AZ Maastricht, The Netherlands; 6 Present Address: Aligos Therapeutics, Leuven, Belgium

**Keywords:** Chemokines, Cytokines, Chemokines, Interleukins, Cardiovascular biology, Gene delivery, Genetic vectors, Immunology, Cardiology

## Abstract

Viral myocarditis (VM) is an important cause of heart failure (HF) in children and adults. However, the molecular determinants involved in cardiac inflammation and cardiomyocyte necrosis remain poorly characterized, and cardioprotective molecules are currently missing. Here, we applied an in vivo method based on the functional selection (FunSel) of cardioprotective factors using AAV vectors for the unbiased identification of novel immunomodulatory molecules in a Coxsackievirus B3 (CVB3)-induced myocarditis mouse model. Two consecutive rounds of in vivo FunSel using an expression library of 60 cytokines were sufficient to identify five cardioprotective factors (IL9, IL3, IL4, IL13, IL15). The screening also revealed three cytokines (IL18, IL17b, and CCL11) that were counter-selected and likely to exert a detrimental effect. The pooled overexpression of the five most enriched cytokines using AAV9 vectors decreased inflammation and reduced cardiac dilatation, persisting at 1 month after treatment. Individual overexpression of IL9, the top ranking in our functional selection, markedly reduced cardiac inflammation and injury, concomitant with an increase of anti-inflammatory Th2-cells and a reduction of pro-inflammatory Th17- and Th22-cells at 14 days post-infection. AAV9-mediated FunSel cardiac screening identified IL9 and other four cytokines (IL3, IL4, IL13, and IL15) as cardioprotective factors in CVB3-induced VM in mice.

## Introduction

Viral myocarditis (VM) is characterized by excessive cardiac inflammation and injury leading to heart failure and sudden death. Cardiotropic viruses can directly cause tissue destruction; nevertheless, the host inflammatory response induces most of the cardiac damage^1^.

To date, specific therapies for VM are missing, besides treating heart failure (HF) when systolic dysfunction is present^2^. Addressing cardiac inflammation with systemic immunosuppression is not possible since it harbors the risk of resurging viral replication. The development of tailored therapy for VM is further hindered by complex patient stratification and knowledge gaps regarding the transition from viral infection to acute myocarditis^3^. Moreover, the increasing evidence of cardiovascular involvement in the COVID19 pandemic has further highlighted the urgent need for treatment options for viral myocarditis^4^.

Cytokines are low-molecular-weight proteins secreted by the inflamed tissue and by infiltrating immune cells. They regulate the inflammatory response and stimulate cell division, proliferation, and differentiation of immune cells through a complex signaling network. These molecules can act as primary pro-inflammatory mediators to ensure pathogen eradication or as factors that resolve inflammation and initiate healing^5^. However, in pathological conditions, the same molecules can trigger detrimental vicious cycles or “cytokine storms”, promoting massive immune cell recruitment and proliferation in the myocardial tissue and conspicuous cardiomyocyte death, severely impairing cardiac function^6^. Due to their pleiotropic nature, their shared signaling pathways, and complex interconnected networks, investigating individual cytokines can underestimate crucial cytokine-mediated communication regulating the immune response distinctive for each pathology^7^.

The molecular factors implicated in the development of excessive cardiac inflammation remain poorly characterized, and soluble cardioprotective molecules are currently missing. Therefore, we hypothesized that targeting soluble factors, such as cytokines, during the acute inflammatory phase and manipulating their expression could reduce cardiac damage and consequent remodeling. To find effective individual cytokines or cytokine combinations exerting a therapeutic effect in VM, here we applied a recently developed Functional Selection (FunSel) procedure allowing the unbiased selection of effective factors in vivo^8^.

## Results

### FunSel identifies IL9 as a potential cardioprotective agent in the sub-acute phase during VM

**Figure 1:**
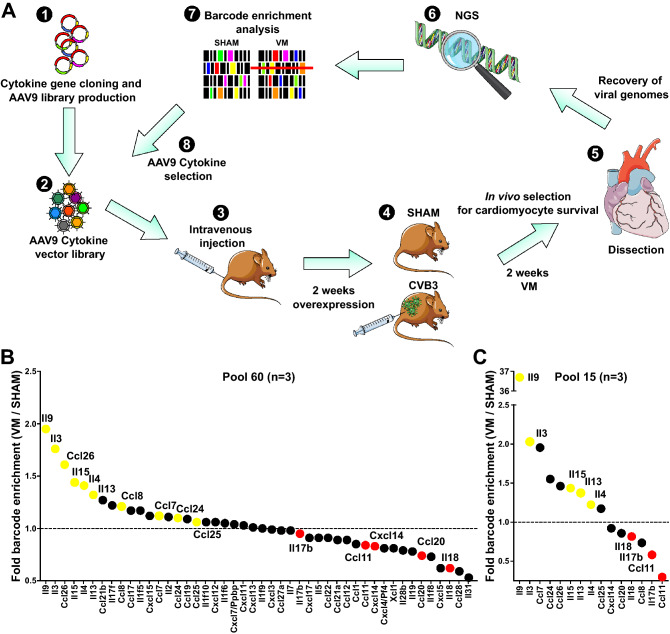
FunSel identifies IL9 as a cardioprotective agent in the sub-acute phase during VM. (**A**) Graphical representation of the events included in the Functional Selection (FunSel) technique to screen potential cardioprotective targets under the selective pressure of viral myocarditis (VM). *NGS* Next-generation sequencing. **B** Results of competitive selection of the most efficiently expressed cytokines (46 of the initial 60) in Pool 60. Each dot represents the relative enrichment of each cytokine vector detected by barcode sequencing, 2 weeks after Coxsackievirus B3 (CVB3)-infection (1.00 = no enrichment, dotted line; > 1.00 = positive selection; < 1.00 = negative selection) from pools of 3 animals per group. From this first set of results, 10 enriched cytokines (in yellow) and 5 depleted cytokines (in red) were selected to obtain Pool 15, with which a second FunSel cycle was performed. (**C**) Same as in (**B**) after overexpression of pool 15, 2 weeks after CVB3-infection from biological pools of 3 animals per group. In yellow, cytokines enriched in VM, chosen to study further (IL9, IL3, IL15, IL13, and IL4); in red, cytokines most consistently depleted in both rounds of VM (IL18, IL17b, and Ccl11).

A schematic diagram of the FunSel technique for the in vivo selection of immunomodulating molecules in VM is shown in Fig. 1A. We generated a targeted AAV vector library using AAV9, which displays cardiotropic properties in mice^9^. The library contained an unbiased selection of interleukins (n = 30) and chemokines (n = 30), most of which are poorly investigated for their involvement in myocarditis. Each vector belonging to this collection included a unique DNA barcode allowing its unequivocal identification in the heart upon next-generation sequencing (NGS). FunSel consisted of a two-round screening process under the selective pressure of VM for 14 days. Vectors that overexpressed cardiomyocyte survival-promoting factors persisted longer in the infected myocardium than vectors expressing detrimental factors (or neutral factors) present in the same library. In FunSel, the selective pressure eventually induces the progressive enrichment of protective vectors, which is detected by analyzing the relative number of sequencing reads for each vector at the end of the process, compared to those in uninfected heart controls. The procedure eventually generates a comparative ranking list of the AAV9 library, based on the relative abundance of each vector in cardiac tissue after VM (3 pooled biological replicates/group, Supplementary Information 1, Table S1).

The ratio between relative AAV barcode frequency between transduced CVB3-infected and sham-control transduced hearts after the first round of in vivo selection with the AAV9 library (Pool 60) is shown in Fig. 1B.

By setting the barcode recovery ratio to the statistically expected value > 0.20, the NGS detected 46 cytokines. Sequencing reads for the remaining 14 cytokines were not deemed reliable, as they were not effectively packaged in Pool 60 (Supplementary Information 2, Fig. S1A–C).

The six most enriched cytokines at the end of the first screening round were IL9, IL3, CCL26, IL15, IL4, IL13. We included these six cytokines in another AAV9 pool (Pool 15) for the second round of FunSel, together with other four cytokines that were also selected (CCL8, CCL7, CCL24, CCL25) and additional five depleted cytokines as controls (IL17b, CCL11, CXCL14, CCL20, 1L18; Table S1). We included depleted factors (and therefore potentially detrimental) in the pool to maintain a competitive selection among the factors in the pool (enriched vs. depleted) and to confirm their negative effect on cardiomyocyte survival upon CVB3-infection.

At the end of the second round of screening with Pool 15 (Fig. 1C), IL9, IL3, IL13, IL15, and IL4 were confirmed to be among the most abundantly recovered interleukins in CVB3-infected hearts (Table S1), with IL9 having the highest enrichment in both pools (1.95- and 36.70-fold change compared to sham controls, respectively; Fig. 1B,C). Factors depleted in the first round were confirmed as depleted in the second round. These included IL18, IL17b, and CCL11, with CCL11, also known as eotaxin^10^, reaching the lowest enrichment level in Pool 15 (0.30-fold change compared to sham controls, Fig. 1C).

Our investigations focused on IL9, IL3, IL13, IL15, and IL4. From the VM-enriched cytokines in Pool 15, we did not consider further CCL7, CCL24, and CCL26 as information of these chemokines is already available in acute myocarditis^11,12^.

### Synergic overexpression of IL9 plus IL3, IL4, IL13, and IL15 (Pool 5) is cardioprotective in the acute phase of VM

First, we investigated whether the synergic expression of the five enriched interleukins (IL-9, IL3, IL4, IL13, IL15; Pool 5) could be cardioprotective during the acute phase of VM and could also result later in beneficial effects against cardiac dysfunction.

**Figure 2:**
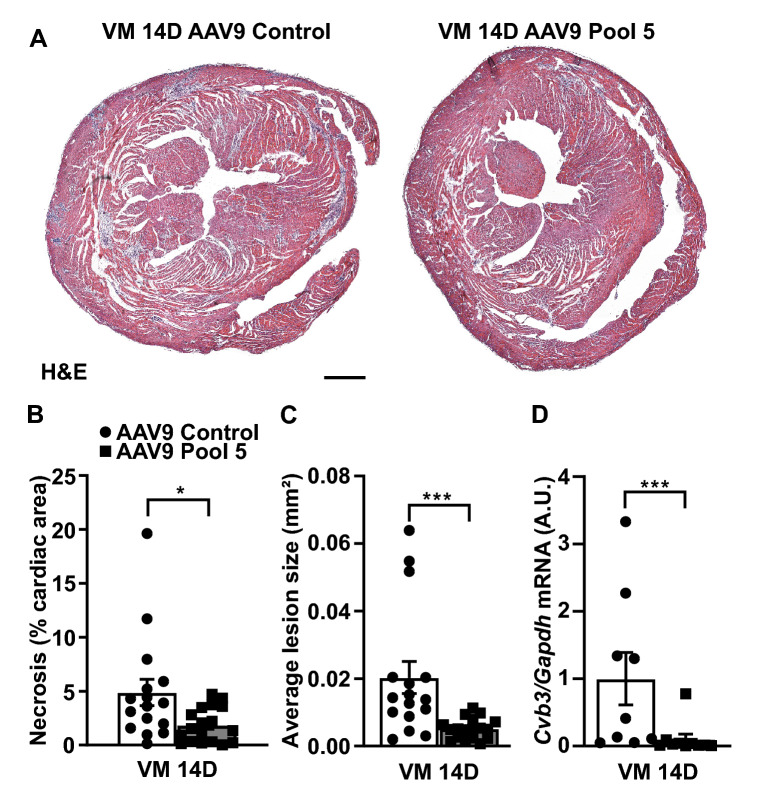
Synergic overexpression of the top listed cytokines (Pool 5) decreases cardiac necrosis in VM. (**A**,**B**) Cardiac necrosis and (**C**) average lesion size detected by hematoxylin and eosin detected by hematoxylin and eosin (H&E) staining 14 days after CVB3 infection in Pool 5-overexpressing hearts and controls. Scale bar = 500 µm. Significance assessed by Mann–Whitney *U* test with *p ≤ 0.05; ***p ≤ 0.001. (**D**) Relative *Cvb3* viral genome expression levels at 14 days post-infection in Pool 5-overexpressing hearts and controls. Significance assessed by Mann–Whitney *U* test with ***p ≤ 0.001. All values are expressed as mean ± SEM.

We administered 1 × 10^11^ viral particles of Pool 5 AAV9 vectors 2 weeks before CVB3 infection. Cardiac expression of all five cytokines was significantly increased over a control AAV9 vector (Supplementary Information 2, Fig. S2A–E). Pool 5 overexpression reduced cardiac necrosis by > 2 folds compared to infected controls (AAV9 Control: 4.9% ± 1.2 vs. AAV9 Pool 5: 1.8% ± 0.4, p < 0.05; Fig. 2A,B), also decreasing the average size of the cardiac necrotic lesions by 74% (AAV9 Control: 0.020 mm^2^ ± 0.005 vs. AAV9 Pool 5: 0.005 mm^2^ ± 0.001, p < 0.001; Fig. 2C), and the levels of CVB3 RNA genomes by 90% (AAV9 Control: 1.00 A.U. ± 0.39 vs. AAV9 Pool 5: 0.10 A.U. ± 0.24, p < 0.001; Fig. 2D).

**Figure 3:**
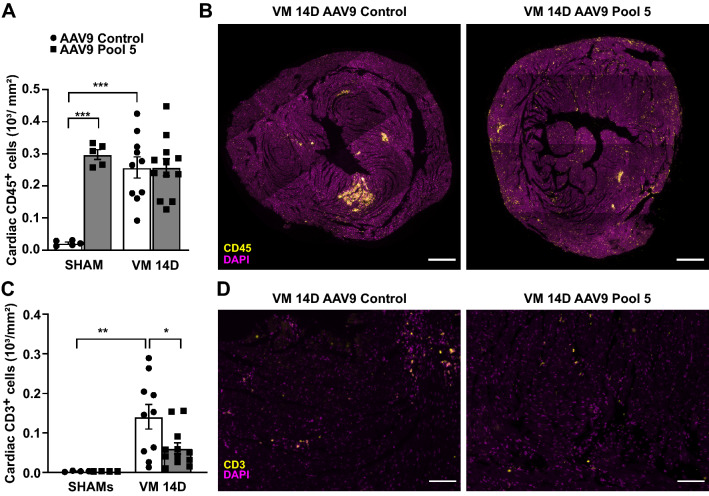
Overexpression of IL9 plus IL3, IL4, IL13, IL15 (Pool 5) decreases T lymphocyte response in the acute phase. (**A**,**B**) CD45 staining (yellow patches, leukocytes) at 14 days post-infection in Pool 5-overexpressing hearts and controls. Scale bar = 500 µm. Significance assessed by two-way ANOVA followed by Tukey’s test with ***p ≤ 0.001. (**C**,**D**) CD3^+^ staining (T lymphocytes) 14 days after CVB3 infection in Pool 5-overexpressing hearts and controls. Scale bar = 100 µm. Significance assessed by two-way ANOVA followed by Tukey’s test with *p ≤ 0.05; **p ≤ 0.01. All values are expressed as mean ± SEM.

There was no difference in the levels of cardiac CD45^+^ leukocytes between Pool 5-overexpressing and AAV9-control animals after CVB3-infection (Fig. 3A,B). However, upon Pool 5 overexpression, the CD45^+^ leukocyte infiltrate in the infected myocardium was diffuse and interstitially distributed rather than concentrated in the necrotic foci (Fig. 3B). Pool 5 significantly reduced cardiac CD3^+^ T lymphocytes in the CVB3-infected myocardium by > 2 folds compared to VM controls (AAV9 Control: 140 cells/mm^2^ ± 31 vs. AAV9 Pool 5: 61 cells/mm^2^ ± 14, p < 0.01; Fig. 3C,D).

**Figure 4:**
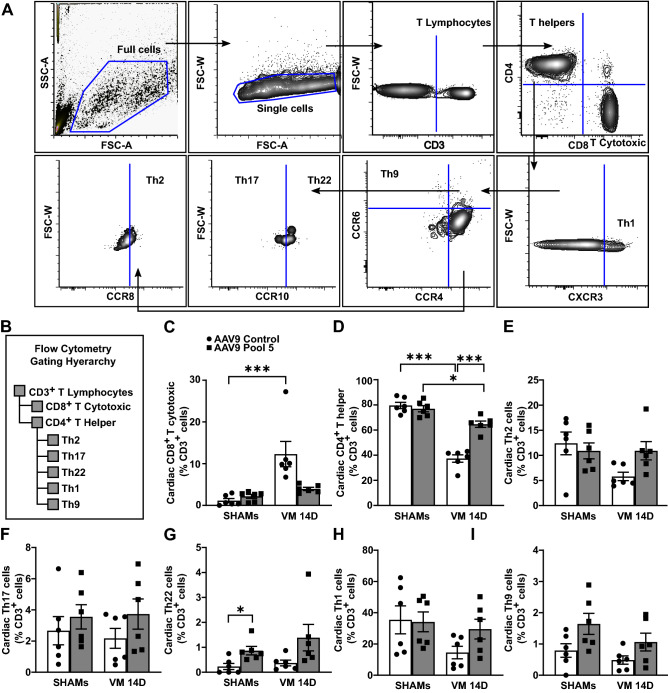
Overexpression of Pool 5 alters cardiac T cytotoxic and T helper lymphocyte balance in the acute phase. (**A**,**B**) Flow cytometry gating strategy and hierarchy to detect T lymphocyte subpopulation at 14 days post-infection in Pool 5-overexpressing hearts and controls. (**C**) T cytotoxic and (**D**) T helper lymphocytes detected by flow cytometry in Pool 5-overexpressing hearts and controls at 14 days post-infection. Significance assessed by a Kruskal–Wallis test (**C**) and a two-way ANOVA (**D**), followed by Tukey’s test with *p ≤ 0.05; ***p ≤ 0.001. (**E**) T helper 2 lymphocytes (Th2), (**F**) Th17, (**G**) Th22, (**H**) Th1, and (**I**) Th9 detected by flow cytometry in infected Pool 5-overexpressing hearts and infected controls 14 days after CVB3 infection. Significance assessed by Kruskal–Wallis test with *p ≤ 0.05 for panels (**E**), (**F**), and (**G**) or two-way ANOVA followed by Tukey’s test for panels (**H**) and (**I**). All values are expressed as mean ± SEM.

Considering the well-known effects of all Pool 5 cytokines on T lymphocyte function^7^, we analyzed cardiac CD3^+^ T lymphocyte subpopulations using flow cytometry (gating strategy depicted in Fig. 4A,B). Cardiac T cytotoxic lymphocytes were decreased although not significantly (AAV9 Control: 12.3% ± 3.0 vs. AAV9 Pool 5: 4.0% ± 0.4, p < 0.001; Fig. 4C), while cardiac T helper cells significantly increased (AAV9 Control: 37.3% ± 3.0 vs. AAV9 Pool 5: 64.6% ± 2.5, p < 0.001; Fig. 4D). Pool 5 overexpression did not change T helper subtype recruitment after CVB3 infection (Fig. 4E–I), except for an increase in Th22 cells in uninfected controls (AAV9 Control: 0.2% ± 0.1 vs. AAV9 Pool 5: 0.9% ± 0.2, p < 0.05; Fig. 4G).

Hence, overexpression of Pool 5 has a beneficial effect in reducing cardiac damage and viral presence in the acute phase of VM, reducing overall cardiac T lymphocyte levels and T cytotoxic lymphocytes while favoring a T helper immune response.

### Pool 5 overexpression reduces systolic cardiac dysfunction in the chronic phase of VM

**Figure 5:**
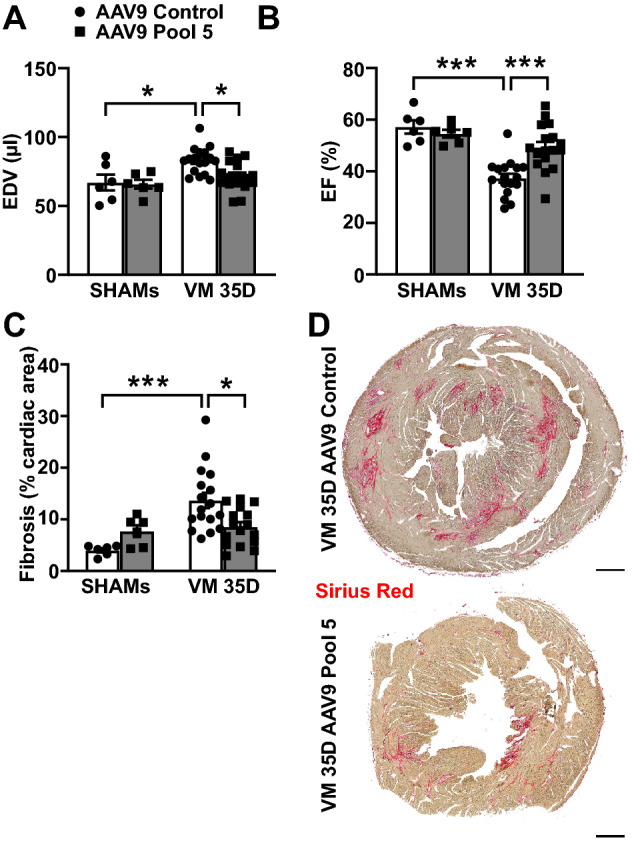
Overexpression of Pool 5 improves cardiac function in the chronic phase during VM. (**A**) End-diastolic volume (EDV) and (**B**) ejection fraction (EF) measured by echocardiography in infected Pool 5-overexpressing C3H mice and infected controls 35 days after CVB3 infection. Significance assessed by two-way ANOVA followed by Tukey’s test with *p ≤ 0.05, ***p ≤ 0.001. (**C**,**D**) Fibrosis detected by Sirius Red staining in infected Pool 5-overexpressing hearts and controls 35 days after CVB3 infection. Scale bar = 500 µm. Significance assessed by two-way ANOVA followed by Tukey’s test with *p ≤ 0.05; ***p ≤ 0.001. All values are expressed as mean ± SEM.

At 5 weeks post-infection, Pool 5 overexpression improved overall cardiac function compared to controls (full echocardiographic analysis in Supplementary Information 2, Table S2). In particular, the presence of the five cytokines prevented cardiac remodeling, significantly decreasing end-diastolic dilatation (end-diastolic volume, EDV, AAV9 Control: 83 µL ± 2 vs. AAV9 Pool 5: 72 µL ± 3, p < 0.05; Fig. 5A) and improving systolic function (ejection fraction, EF, AAV9 Control: 37% ± 2 vs. AAV9 Pool 5: 49% ± 2, p < 0.001; Fig. 5B) upon CVB3-infection. In line with the functional improvement, cardiac Sirius Red-labelled fibrosis was significantly reduced (AAV9 Control: 13.7% ± 1.5 vs. AAV9 Pool 5: 9.3% ± 1.1, p < 0.05; Fig. 5C,D).

Collectively, these results are consistent in showing that the combined cardiac overexpression of IL9, IL3, IL4, IL13, and IL15 during the chronic phase of CVB3-induced VM can significantly improve the outcome of cardiac disease, reducing cardiac dilatation and dysfunction.

### IL9 overexpression prevents necrosis and improves immune response in the acute phase of myocarditis

**Figure 6:**
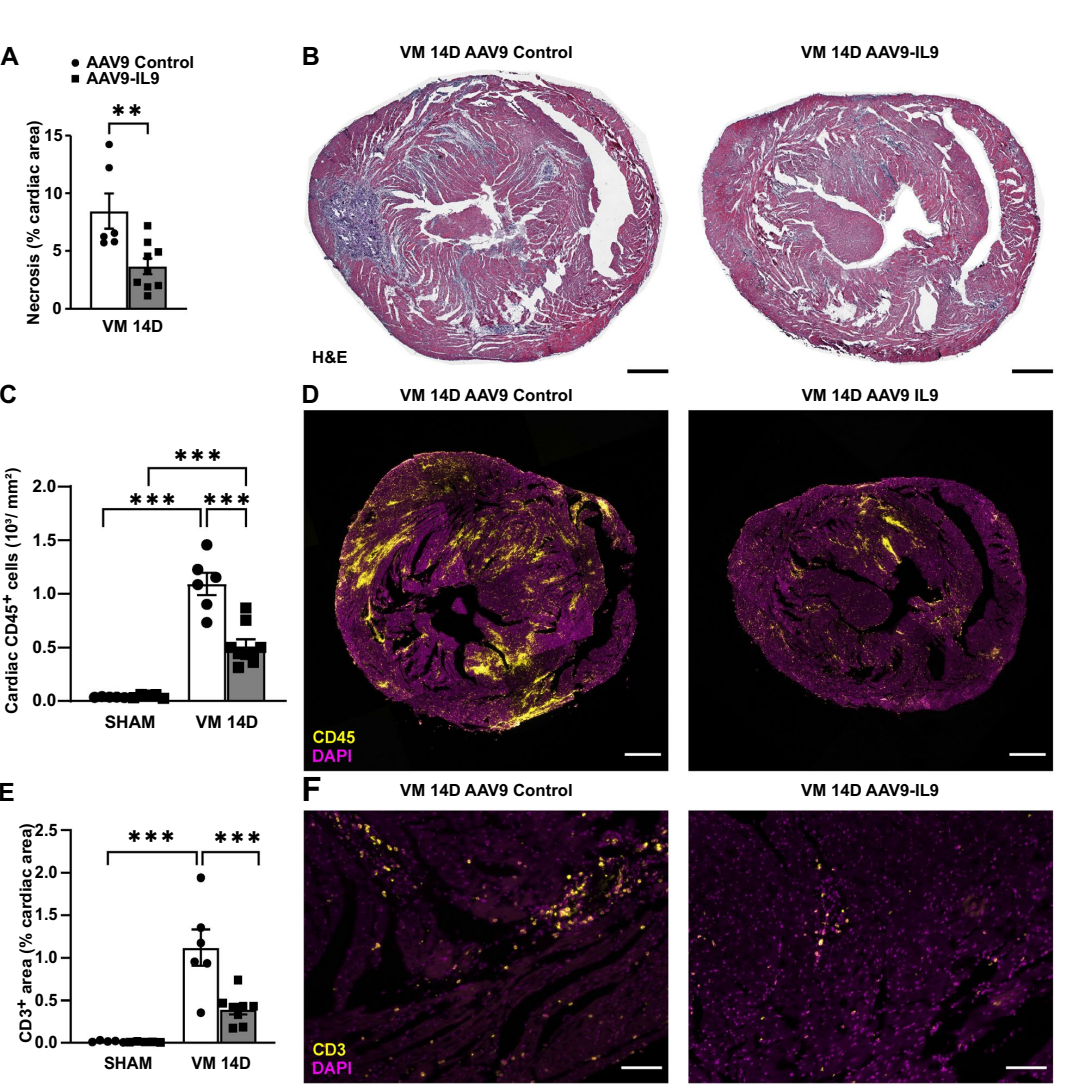
IL9 overexpression improves VM outcome, decreasing T lymphocyte response in the acute phase. (**A**,**B**) Cardiac necrosis detected by hematoxylin and eosin (H&E) staining 14 days after CVB3 infection in IL9-overexpressing hearts and controls. Scale bar = 500 µm. Significance assessed by Mann–Whitney *U* test with **p ≤ 0.01. (**C**,**D**) CD45 staining (leukocytes) in infected IL9-overexpressing and infected control hearts at 14 days post-infection. Scale bar = 500 µm. Significance assessed by two-way ANOVA followed by Tukey’s test with ***p ≤ 0.001. (**E**,**F**) CD3 staining (T lymphocytes) in infected IL9-overexpressing and infected control hearts 14 days after CVB3 infection. Scale bar = 100 µm. Significance assessed by two-way ANOVA followed by Tukey’s test with ***p ≤ 0.001. All values are expressed as mean ± SEM.

AAV9-IL9 showed remarkable enrichment over all other interleukins in both Pool 60 (Fig. 1B) and Pool 15 (Fig. 1C), and in combination with IL3, IL4, IL13, and IL15 (Pool 5), significantly improved CVB3-induced myocarditis (Figs. 2 and 5). Therefore, we focused our attention on this factor as a single treatment to VM. We administered AAV9-IL9 or a control AAV9 vector 2 weeks before CVB3 infection and then assessed mice 2 weeks post-CVB3 infection. We observed a 25-fold change in IL9 cardiac expression compared to controls (AAV9-Control: 1.0 ± 0.2 vs. AAV9-IL9: 25.2% ± 5.8, p < 0.001; Supplementary Information 2, Fig. S3A). IL9 overexpression induced a 57% reduction in cardiac necrosis (AAV9 Control: 8.5% ± 1.5 vs. AAV9-IL9: 3.7% ± 0.7, p < 0.01; Fig. 6A,B) and was associated with a 52% reduction of infiltrating CD45^+^ leukocytes in the cardiac tissue (AAV9 Control: 1043 cells/mm^2^ ± 103 vs. AAV9-IL9: 514 cells/mm^2^ ± 60, p < 0.001; Fig. 6C,D). The number of CD68^+^ macrophages (Supplementary Information 2, Fig. S3B,C) and the levels of CVB3 RNA genomes (Su, Fig. S3D) did not change significantly.

IL9 overexpression also induced a nearly threefold reduction in CD3^+^ T lymphocytes at 14 days after CVB3 infection compared to controls (AAV9-Control: 1.2% CD3-stained cardiac area ± 0.2 vs. AAV9-IL9: 0.4% CD3-stained cardiac area ± 0.1, p < 0.001; Fig. 6E,F).

**Figure 7:**
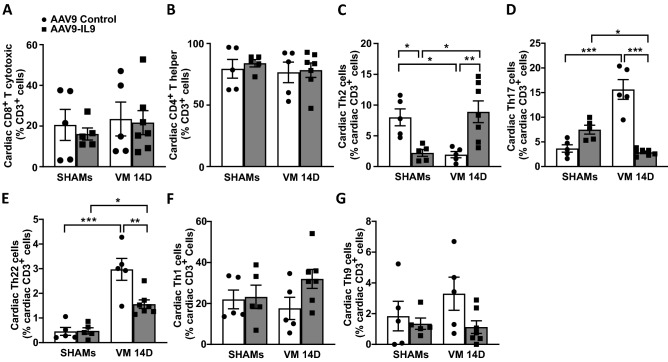
IL9 overexpression alters T helper subpopulations, enhancing Th2 and reducing Th17/22 presence in the acute phase. (**A**) T cytotoxic and (**B**) T helper lymphocytes detected by flow cytometry in IL9-overexpressing hearts and controls at 14 days post-infection. Significance assessed by two-way ANOVA, followed by Tukey’s test. (**C**) T helper 2 lymphocyte (Th2), (**D**) Th17, (**E**) Th22, (**F**) Th1, and (**G**) Th9 cardiac subpopulations detected by flow cytometry in CVB3-infected IL9-overexpressing hearts and infected controls at 14 days post-infection. Significance assessed by two-way ANOVA followed by Tukey’s test or by Kruskal–Wallis test (**F**), with *p ≤ 0.05, **p ≤ 0.01, ***p ≤ 0.001. All values are expressed as mean ± SEM.

IL9 overexpression did not alter the ratio between T cytotoxic and T helper among CD3^+^ T lymphocytes (Fig. 7A,B), but markedly increased the percentage of cardiac anti-inflammatory T helper 2 (Th2) lymphocytes (AAV9 Control: 2.0% ± 0.5 vs. AAV9-IL9: 8.9% ± 1.8, p < 0.01; Fig. 7C). IL9 overexpression also drastically decreased the percentage of T helper 17 (Th17; AAV9 Control: 15.6% ± 2.0 vs. AAV9-IL9: 2.9% ± 0.3, p < 0.001; Fig. 7D) and T helper 22 (Th22; AAV9 Control: 3.0% ± 0.5 vs. AAV9-IL9: 1.6% ± 0.2, p < 0.01; Fig. 7E) lymphocytes, both associated with adverse cardiac inflammation and remodeling^13,14^. T helper 1 or T helper 9 lymphocyte percentages did not change upon IL9 overexpression in VM (Fig. 7F,G).

**Figure 8:**
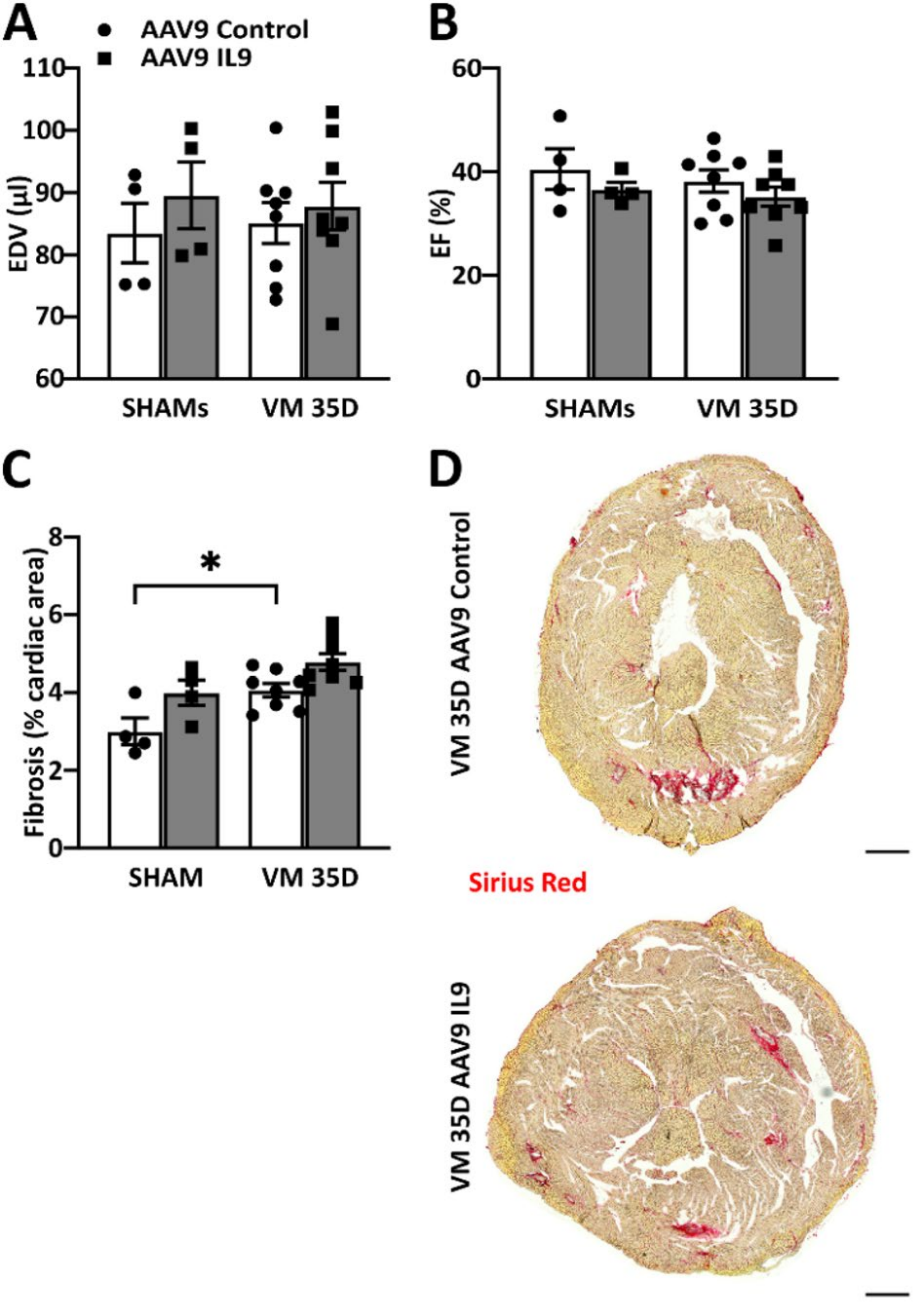
IL9 overexpression is not sufficient to improve cardiac function in the chronic phase during VM. (**A**) End-diastolic volume (EDV) and (**B**) ejection fraction (EF) measured by echocardiography in infected IL9-overexpressing C3H mice and infected controls 35 days after CVB3 infection. Significance assessed by two-way ANOVA, followed by Tukey’s test. (**C**,**D**) Fibrosis detected by Sirius Red staining in infected IL9-overexpressing hearts and controls 35 days after CVB3 infection. Scale bar = 500 µm. Significance assessed by two-way ANOVA, followed by Tukey’s test with *p ≤ 0.05. All values are expressed as mean ± SEM.

Despite the beneficial effect on T lymphocyte recruitment and necrosis at 14 days, the individual overexpression of IL9 did not significantly change cardiac dimensions or systolic function at 35 days post-infection (Fig. 8A,B, complete echocardiographic analysis in Supplementary Information 2, Table S3), although uninfected controls in both groups showed lower than expected ejection fractions, possibly due to anesthesia levels that also produced lower heart rates than in other experiments. Consistently, at 35 days post-infection, there was no significant difference in cardiac fibrosis (Fig. 8C,D).

Collectively, these findings indicate that IL9 overexpression increases the cardio-protective Th2 response in CVB3-myocarditis, decreasing at the same time the detrimental Th17 and Th22 populations at 14 days. However, IL9 alone is insufficient to determine long-term beneficial effects on cardiac function and prevent adverse cardiac remodeling.

## Discussion

There is a definite need to develop novel therapies for viral myocarditis. Current treatments for this condition are only aimed to prevent or treat systolic dysfunction and arrhythmias and are essentially based on pathophysiological principles defined decades ago. Of note, none of the available drugs are specific for this condition, no biologic treatment is available (all the drugs are small molecules), and, most relevant, none of the therapies is aimed to target the pathological inflammatory response underlying myocarditis. In this study, we, therefore, wanted to address the issue of identifying an effective biological treatment that could specifically prevent cardiac damage, induced by the virus or by the immune response, in the context of viral myocarditis.

FunSel is a novel procedure^8^ based on the in vivo selection of tissue-protective factors irrespective of an a priori knowledge of their physiological mechanisms of action, expression levels or localization. Pools of barcoded AAV vectors coding for secreted factors (to expand their protective function beyond the transduced cell) are used to transduce the heart. In principle, each vector delivers its transgene to a different cardiomyocyte. In practice, co-infection of the same cell by more than one vector, and thus co-selection, can occur. However, these events do not prevent the identification of cardioprotective factors, but simply lowers the quantitative difference between selected and non-selected factors, without altering the ranking of the factors in the pool, as previously validated^8,15^. In this study viral infection and consequently cardiac inflammation act as a selective challenge, promoting an in vivo competitive process between putative beneficial and detrimental or non-effective genes within the same vector pool. Transduced cardiomyocytes dying as a consequence of viral infection or immune-mediated cytotoxicity are rapidly removed from the myocardium, therefore, after 2 weeks of VM, DNA coding for neutral/detrimental factors are no longer detectable in the surviving cells. Barcodes from the surviving cardiomyocytes are recovered, and their relative abundance is revealed by a two-step PCR approach followed by next-generation sequencing, without introducing any bias, as previously validated^15^. Enriched vectors are expected to express protective proteins. FunSel is unique in permitting functional (as opposed to phenotypic) selection directly in vivo, is unbiased in terms of knowledge of the mechanism of action of the factors, and is competitive among the factors, eventually permitting their ranking for efficacy.

In this study, the pooled AAV9-mediated overexpression of 60 cytokines in a human Coxsackievirus-B3-induced myocarditis mouse model allowed the identification of five cardioprotective interleukins (IL9, IL3, IL4, IL13, and IL15). The individual, AAV9-mediated overexpression of IL9, the most enriched cytokine in the screening, significantly decreased cardiac inflammation and necrosis 14 days after CVB3 infection. Along with decreased T lymphocyte recruitment, IL9 increased the number of cardiac anti-inflammatory Th2 lymphocytes while reducing detrimental Th17 and Th22 lymphocytes at 14 days.

IL9 is a pleiotropic cytokine and member of type II immune response, recently associated with the pathogenesis of inflammatory diseases and the preservation of immune tolerance^16,17^. The observed anti-inflammatory properties of IL9 overexpression are consistent with findings in IL9 knockout mice, in which its absence exacerbates cardiac inflammation and injury in VM^18^. We further explored T lymphocyte subpopulations upon IL9 overexpression. IL9 increased the number of cardiac Th2 lymphocytes, while it decreased Th17 and Th22 cells. Th2 lymphocytes may induce a humoral response towards viral infection that dampens adverse cardiac inflammation^19^, which occurs preferentially in female mice, resistant to VM^20^. Th2-secreted cytokines, such as IL4 and IL13 (both also enriched in our FunSel experiments), are crucial anti-inflammatory mediators by increasing the regulatory T (T Reg) lymphocytes and alternatively activated macrophage (M2) during the resolution phase of cardiac inflammation^21^. On the contrary, Th17 lymphocytes are detrimental in VM and may facilitate systolic cardiac dysfunction^22^.

A more persistent effect on cardiac fibrosis and systolic function at 35 days post-infection required the combined expression of IL9 with IL3, IL4, IL13, and IL15. IL9 is known to induce fibrosis in the chronic progression of several pathologies such as heart failure, and pulmonary and hepatic inflammatory diseases^23–25^, through the release of profibrotic tumor growth factor (TGF)^18,23,26^ and connective tissue growth factor (CTGF)^27^. This may explain why prolonged IL9 overexpression, which is inevitable due to the persistence of AAV vectors, did not improve cardiac systolic function or fibrosis at 35 days when administered alone, despite the beneficial effect on cardiac inflammation and necrosis during acute VM at 14 days.

The selection of IL3, IL4, IL13, and IL15 by FunSel after VM selective pressure is in line with the established effects of these cytokines in other experimental settings. IL3 is protective against inflammatory arthritis^28^, while IL3 and IL4 synergistically reduce adverse inflammation by enhancing Th2 lymphocyte function^29^. Individually, IL4 is known to be cardioprotective in VM^30,31^, also enhancing natural killer (NK) anti-viral property^32^. IL13 attenuates cardiac damage, inducing anti-inflammatory (M2) macrophage differentiation and reducing fibrosis^33,34^. IL15 exert a direct anti-apoptotic function on cardiomyocytes^35^ and cardioprotective activity in CVB3-induced myocarditis, reducing T lymphocyte and proinflammatory macrophage (M1) cardiac infiltration^36^. Thus, FunSel appears to have selected molecules endowed with therapeutic potential and have ranked them according to their individual potency.

The factors that were negatively selected in the screening also deserve attention. In the two screening experiments, IL17b, IL18, and CCL11 were consistently among the negatively enriched cytokines in the VM hearts, suggesting a possible detrimental role in cardiomyocyte survival during VM. IL17b is a homolog of IL17a, and both cytokines are signature factors of Th17^37^. IL17b stimulates the release of tumor necrosis factor (TNF) and IL1β and promotes heart failure in human myocarditis^13^. IL18 is a pro-inflammatory, IFNγ-inducing cytokine released by cardiomyocyte pyroptosis during cardiovascular diseases^38^ and has been associated with cardiac inflammation during myocarditis induced by the encephalomyocarditis virus and by *Trypanosoma* infection^39,40^. Finally, CCL11, also known as eotaxin-1, is one of the main eosinophil and mast cell chemoattractants in cardiac fibrosis and mast cell activation in the host-to-graft response^10^. Thus, FunSel again appears to have appropriately selected against factors potentially exerting adverse effects also in VM.

## Conclusions

Collectively, our data show that FunSel is an efficient method for the in vivo selection of immunomodulatory molecules that provide a survival advantage to myocardial tissue upon VM, specifically oriented to the acute phase of the disease when most of the T lymphocyte-related necrosis occurs. In terms of translation of these findings to therapy, it is worth noting that the few immunomodulating therapies that have reached clinical trials so far, including IL1β-blocking therapy^41^, or, more recently, IL1 receptor antagonist (IL1ra) administration^42^, are all based on the delivery of antagonists to potentially detrimental factors. Cytokines exerting a positive effect, such as IL9, can equally be considered for administration, either in the form of recombinant proteins or through the delivery of their mRNAs. This can be performed directly into the heart upon coronary catheterization or into the skeletal muscle for systemic protein production. The possibility of using the Stable Nucleic Acid Lipid Particle (SNALP) technology for the nanoformulation of modified mRNAs, as in the recent COVID-19 vaccines^43,44^, to achieve transient factor expression is an exciting therapeutic possibility for VM, which warrants further investigation.

## Methods

### Sources

All key resources and materials (including experimental animals, cell lines, antibodies and tools) are listed in Supplementary Information 3, Table S4, including manufacturer (when applicable), catalogue number, and Research Resource Identifiers (RRIDs) when applicable.

### Generation of the AAV plasmid library

The plasmid library was generated as previously described^8,15^. Briefly, all cDNAs were individually cloned into a modified pZac2.1 vector named pGi, under the control of the constitutive cytomegalovirus immediate-early (CMV-IE) promoter. At the 3’-end of each cDNA, a unique 10-bp sequence (barcode) was inserted, allowing detection upon barcode PCR amplification and sequencing. Barcodes differed for at least two base pairs to avoid errors during sequencing.

### Production and purification of recombinant AAV (rAAV) vectors

The rAAV vectors used in this study (http://www.icgeb.org/avu-core-facility) were produced as previously described^8,15^. Briefly, infectious AAV9 vector particles were generated in HEK293T cells, using a dual-plasmid co-transfection system for packaging. Viral stocks were obtained by CsCl (Sigma Aldrich, Belgium) gradient centrifugation, and rAAV titers were determined by measuring the copy number of viral genomes in pooled, dialyzed gradient fractions using Real-Time PCR. The vectors used in this study express the cloned cDNAs under the control of the constitutive cytomegalovirus immediate-early (CMV-IE) promoter, and all the viral stocks had titers between 10^12^ and 10^13^ viral genome (vg) per mL.

### Animal experiments

#### General informations

Whether possible, a priori sample size was determined using G*Power (3.1.9.4)^45^ considering an F-test (ANOVA: fixed effects omnibus), an effect size f = 0.98, a level of significance α = 0.05 and a power of 0.80 in a four groups-settings.

All the animals were randomly allocated to the groups prior to unequivocal identification by P.C. No other confounding factors were taken into consideration. During animal allocation and experimental procedure no blinding procedure was implemented. During outcome assessment and data analysis a blinding procedure was performed, temporary erasing animal allocation within the experimental groups.

#### In vivo AAV9 library screening

The AAV9 vector library and enrichment analysis have been performed as previously published^15^. Briefly, equimolar plasmid pools were used for the correspondent AAV9 vector production. Each vector preparation (100 µL, containing 5 × 10^10^ viral genomes, vg) was injected intravenously into 3-to-4-week-old commercial C3H/HeNHsd male mice (n = 12/group, Envigo, Netherlands). Two weeks later, animals were intraperitoneally injected with a bolus of 10^7^ CCID_50_ (cell culture 50% infective dose) of CVB3 Nancy strain (VR-30, ATCC, USA) as previously described^46^ or with 0.9% NaCl sterile solution.

After 14 dpi, the animals were euthanized, and cardiac DNA was extracted using the DNeasy Blood and Tissue kit (Qiagen, Belgium) from at least three animals per group and pooled in an equimolar amount. The DNA barcodes from the extracted DNA were amplified by two subsequent cycles of polymerase chain reaction (PCR) amplification, first with primers annealing to common regions on the plasmid vector (pGi Fw and pGi Rv, Supplementary Information 3, Table S4) and then with primers containing an Illumina index (Fw Illumina and True Seq adapter Index Rv, Supplementary Information 3, Table S4) required for sample multiplexing. Amplicons were purified from agarose gel and were sequenced on a HighSeq 2000 Illumina instrument. Next-generation sequencing (NGS) generated at least 1 million reads per sample, and samples with a number of reads < 5 were excluded from the analysis. An algorithm developed using the R software (version 4.0.4) allowed to assign the number of reads of each barcode to the correspondent gene name, considering an error of one bp to assign a correct barcode for unequivocal clone identification. For both Pool 60 and Pool 15, the barcode reading ratio in the original AAV pool lysate and the cardiac tissue was compared to the corresponding expected presence (1/60 and 1/15, respectively). Vectors with a barcode recovery ratio lower than 0.20 from any biological source were discarded from further analysis as not efficiently packaged (injected lysate) or not efficiently overexpressed vectors, respectively (Supplementary Information 2, Fig. S1A–C). For all the other clones, an enrichment ratio between the CVB3-infected heart and not-infected cardiac tissue was determined. Factors with a ratio > 1.00 were considered positively enriched in CVB3-infected hearts. Those with a ratio < 1.00 were considered depleted in CVB3-infected hearts. The factors with a ratio equal to 1.00 were considered uninfluential. The screening cycle was repeated twice, first with Pool 60 and then with Pool 15.

#### Pool 5 and IL9 overexpression

For the analysis of AAV9 Pool 5 effects, 100 µL of combined recombinant AAV vectors (AAV9 control or AAV9 Pool 5; 1 × 10^11^ vg per animal) were injected intravenously into 3-to-4-week-old commercial C3H/HeNHsd male mice (n = 52 AAV9 control; n = 52 AAV9 Pool 5; Envigo, Netherlands). After a 2-week overexpression period, all animals were injected intraperitoneally with a bolus of 10^7^ CCID_50_ of CVB3 Nancy strain or with 0.9% NaCl sterile solution. Mice were either euthanized at 14 dpi for histology and molecular analysis (n = 5 AAV9 control SHAM; n = 12 AAV9 control VM; n = 5 AAV9 Pool 5 SHAM; n = 12 AAV9 Pool 5 VM), or for histology and flow cytometry analysis (n = 6 AAV9 control SHAM; n = 6 AAV9 control VM; n = 6 AAV9 Pool 5 SHAM; n = 6 AAV9 Pool 5 VM), or at 35 dpi for cardiac function and histology (n = 6 AAV9 control SHAM; n = 17 AAV9 control VM; n = 6 AAV9 Pool 5 SHAM; n = 17 AAV9 Pool 5 VM). Two animals in the AAV9 control VM group were found dead during the first timepoint and therefore excluded from further analysis.

For the analysis of AAV9 IL9 effects, the same procedure was adopted: 100 µL of recombinant AAV vectors (AAV9 control or AAV9 IL9; 1 × 10^9^ vg/animal) was injected intravenously into 3-to-4-week old commercial C3H/HeNHsd male mice (n = 34 AAV9 control; n = 38 AAV9 IL9, Envigo, Netherlands). After a 2-week overexpression period, all animals were injected intraperitoneally with a bolus of 10^7^ CCID_50_ of CVB3 Nancy strain or with 0.9% NaCl sterile solution. Mice were euthanized at 14 dpi for histology and molecular analysis (n = 5 AAV9 control SHAM; n = 6 AAV9 control VM; n = 5 AAV9 IL9 SHAM; n = 9 AAV9 IL9 VM), or flow cytometry (n = 5 AAV9 control SHAM; n = 6 AAV9 control VM; n = 5 AAV9 IL9 SHAM; n = 7 AAV9 IL9 VM), or at 35 dpi for cardiac function (n = 4 AAV9 control SHAM; n = 8 AAV9 control VM; n = 4 AAV9 IL9 SHAM; n = 8 AAV9 IL9 VM). One animal in the AAV9 control VM group was found dead during the first timepoint and therefore excluded from further analysis.

Mice were euthanized by a ketamine/xylazine overdose, followed by thoracotomy and cardiac excision. All the hearts collected were washed and exposed to a 1 M KCl solution to induce a homogenous end of cardiac activity in diastole.

### Histology and immunofluorescence

Cardiac tissue was fixed in a Tris-based buffer with zinc ions (BD, Belgium) for at least 48 h at room temperature and processed for further standard histological staining. Paraffin-embedded cardiac sections (4–5 µm) were either stained with Harris-hematoxylin (VWR, Belgium) and Eosin (Sigma Aldrich, Belgium) to evaluate cardiac necrosis. Cardiac tissue was stained with a 0,1% Sirius Red (SR) F 3B (Sigma-Aldrich, Belgium) solution in 4% Picric Acid (Sigma-Aldrich, Belgium) to evaluate cardiac fibrosis.

All antibodies mentioned in this paragraph are listed in Supplementary Information 3, Table S4. Sections were immunolabeled with anti-mouse CD45 (1:100 dilution) for overall inflammation, CD68 (1:100 dilution) for macrophages and CD3 (1:50 dilution) for T lymphocyte recruitment. Tyramide signal amplification (Thermofisher Scientific, Belgium) was used for the CD45 and CD3 staining. In all fluorescent-based immuno-labeled sections, nuclei were counterstained with a 2.5 ng/mL DAPI solution (Sigma-Aldrich, Belgium).

### Microscopy and quantification

All the microscopic images were acquired on an Axiovert 200M microscope (Zeiss, Oberkochen, Germany). For entire heart sections, a series of images taken at 5 × magnification were automatically assembled into one larger image using Zeiss’ mosaic function. For immunophenotyping, images were acquired either at a magnification of 20 × or 40 ×, with 15–20 images per sample. All images were acquired using Zeiss proprietary software Axiovision (Rel. 4.8.2) and analyzed with Fiji distribution of the ImageJ software (version 1.53c)^47^.

Myocardial necrosis was determined as a percentage of the sum of the necrotic areas per total cardiac area, with the aid of a 500,000 µm^2^/square grid centered on the image.

Immunostainings were quantified per myocardial cross-sectional area, as previously described^48^. Briefly, immune cell phenotyping was either determined as the number of CD45 (leukocytes), CD68 (monocytes), CD3 (T lymphocyte) positive cells per cardiac area, or as the percentage of positive-stained area per cardiac area when single-cell detection was not possible. Cardiac fibrosis was determined as the percentage of Sirius Red-stained collagen-positive area per cardiac area, with the aid of the color-threshold tool in ImageJ. A blinded observer (P.C.) performed both imaging and analysis.

### Gene expression analysis

RNA was extracted from cardiac tissues and peritoneal exudates using the RNeasy Fibrous Tissue Mini Kit (Qiagen GmbH, Germany) following the manufacturer’s instructions. For RT-PCR analysis, 1 µg of total RNA was reverse-transcribed into cDNA, using the Qiagen Quantitect^®^ Reverse transcription kit (Qiagen GmbH, Germany), following the manufacturer’s instructions, and transcript levels were quantified using IQ SYBR-green supermix (Bio-Rad, UK) in a MyIQ iCycler (Bio-Rad, UK). Primers (Supplementary Information 3, Table S4) were designed to span exon–exon junctions of the genes of interest. In the case of non-detection datapoints of the corresponding animals were not plotted.

### Transthoracic echocardiography and analysis

Cardiac function was assessed 35 days post-infection using an MS400 (18–38 MHz) transducer on a VEVO 2100 echocardiographer (Fujifilm VisualSonics, Netherlands), as previously described in other animal models^49^. Mice were anesthetized with 5% inhaled isoflurane (Cuphar, Oostkamp, Belgium) followed by 1.5% inhaled isoflurane to maintain them anesthetized. Animals were placed in a supine position on a heated pad to maintain the core body temperature between 37.5–37.7 °C, measured using a rectal probe. Throughout the procedure, both heart and respiratory rates were monitored continuously. Anterior (AW) and posterior wall (PW) thickness, and LV internal diameter (LVID) were assessed on a 2D M-Mode mid-ventricular short-axis (SAX) image. Cardiac output (CO), ejection fraction (EF), and endocardial volume were calculated automatically by the software. For all measurements, at least three stable cardiac cycles were analyzed and averaged. A blinded observer (P.C.) performed the analysis.

### Cardiac immune population isolation

Mice were euthanized by ketamine/xylazine overdose, the chest cavity was exposed, and the right atrium was incised to allow blood outflow. The heart was immediately perfused with 5 mL ice-cold 0.05% EDTA in PBS into the left ventricle to wash away any residual blood from the cardiac vasculature and then excised. Both atria were removed, and the ventricles were placed in ice-cold PBS to isolate the immune cell cardiac population. The ventricles were minced into 1 mm^3^-size piece with a sterile surgical blade and placed in an enzymatic solution containing 2.5 mg/mL collagenase (Sigma-Aldrich, Belgium) for 40 min at 37 °C. After trituration, the single-cell suspension was layered on top of the Histopaque 1083 solution (Sigma-Aldrich, Belgium) to separate the immune from the non-immune fraction. The interphase, containing the immune fraction, was then rinsed with PBS. Blood samples underwent three cycles of erythrocyte lysis instead: specifically, blood was incubated 3’ in ice with an Erylysis buffer (7.3 pH 0.15 M NH_4_Cl, 0.01 M NaHCO_3,_ and 0.1 mM EDTA in PBS) and washed with PBS afterward. Cells were centrifuged at 1600 RPM, and the supernatant was discarded for each lysis round. Finally, all the cell pellets were resuspended in 100 µL of flow cytometry buffer (0.1% BSA and 2 mM EDTA in PBS), and cells were counted. All the samples underwent further processing for flow cytometry analysis.

### Flow cytometry analysis

The immune cell samples were transferred on a V-bottom 96-well plate and blocked with Fc block (anti-mouse CD16/CD32, 1:100 dilution, Thermofisher Scientific, Belgium) for 30 min on ice in the dark. Afterward, the samples were washed with flow cytometry buffer and centrifuged at 1600 RPM at 4 °C for 3 min. The pellets were then stained with the fluorochrome-conjugated antibodies for 30 min on ice in the dark. Afterward, the samples were washed with flow cytometry buffer and centrifuged at 1600 RPM at 4 °C for 3 min. Finally, all the samples were resuspended in 200 µL of flow cytometry buffer and analyzed on a CANTO II flow cytometer (Becton-Dickinson Benelux, Belgium). All antibodies used in this procedure were used at 1:100 dilution/10^6^ cells stained and are listed in the Supplementary Information 3, Table S4.

Cells were gated on singlets and T lymphocyte (CD3^+^ cells) subpopulations were defined as follows: T cytotoxic (CD3^+^, CD8^+^, CD4^−^), T helper (CD3^+^, CD8^−^, CD4^+^), T helper 1 (CD3^+^, CD4^+^, CXCR3^+^), T helper 2 (CD3^+^, CD4^+^, CXCR3^−^, CCR6^−^, CCR4^+^, CCR8^+^), T helper 9 (CD3^+^, CD4^+^, CXCR3^−^, CCR6^+^, CCR4^−^), T helper 17 (CD3^+^, CD4^+^, CXCR3^−^, CCR6^+^, CCR4^+^, CCR10^−^), T helper 22 (CD3^+^, CD4^+^, CXCR3^−^, CCR6^+^, CCR4^+^, CCR10^+^). Data were analyzed using FCS Express 7 (DeNovo Software, California, USA) by a blinded observer (P.C.) and expressed as a percentage of CD3^+^ cells.

### Quantification and statistical analysis

All statistical analyses were performed using GraphPad Prism 9 (GraphPad Software, USA). Data are expressed as mean ± SEM. The normality of the distribution of all continuous variables was assessed using the Shapiro and Wilk test. When comparing only two groups, an unpaired Student *t* test was used if groups passed the normality test, while a Mann–Whitney *U* test was used otherwise. When comparing two variables simultaneously (including treatment and pathology), a 2-way ANOVA with Tukey’s correction was used if groups passed the normality test, while a Kruskal–Wallis test was used otherwise. Graphical data was shown as mean values with error bars indicating the SEM. Two-sided p-value p values of < 0.05 (*), < 0.01 (**) or < 0.001 (***); indicated significant differences between groups.

### Ethics approval and consent to participate

All experiments described in this study are reported in accordance with the ARRIVE guidelines (ARRIVE checklist included). All experiments involving the use of animals or isolation of primary cells from animals were monitored and approved by the KU Leuven Animal Ethics Committee, according to the Belgian law and the guidelines from Directive 2010/63/EU of the European Parliament, under the protocol n. 220/2015. Consent to participate not applicable.

### Consent for publication

All authors have read and approved the manuscript.

## Supplementary Information

Supplementary Table S1.

Supplementary Figures.

Supplementary Table S4.

Supplementary Information.

## Data Availability

All data generated or analyzed during this study (datapoints, means and standard deviations) are included in this published article, and its Supplementary Information files (Supplementary Information 4). All unique rAAV vectors produced in this study are available upon request at the AAV Vector Unit at ICGEB Trieste (http://www.icgeb.org/avu-core-facility/). All key resources and materials are listed in Supplementary Information 3, Table S4.
