# Supplementary material for: AAV9-mediated functional screening for cardioprotective cytokines in Coxsackievirus-B3-induced myocarditis
Source: Sci Rep. 2022 May 4;12:7304. doi: 10.1038/s41598-022-11131-w (PMC9067557; doi:10.1038/s41598-022-11131-w)
Supplement: Supplementary file 2 — Supplementary Figures. [file 41598_2022_11131_MOESM2_ESM.pdf]

Supplementary Data

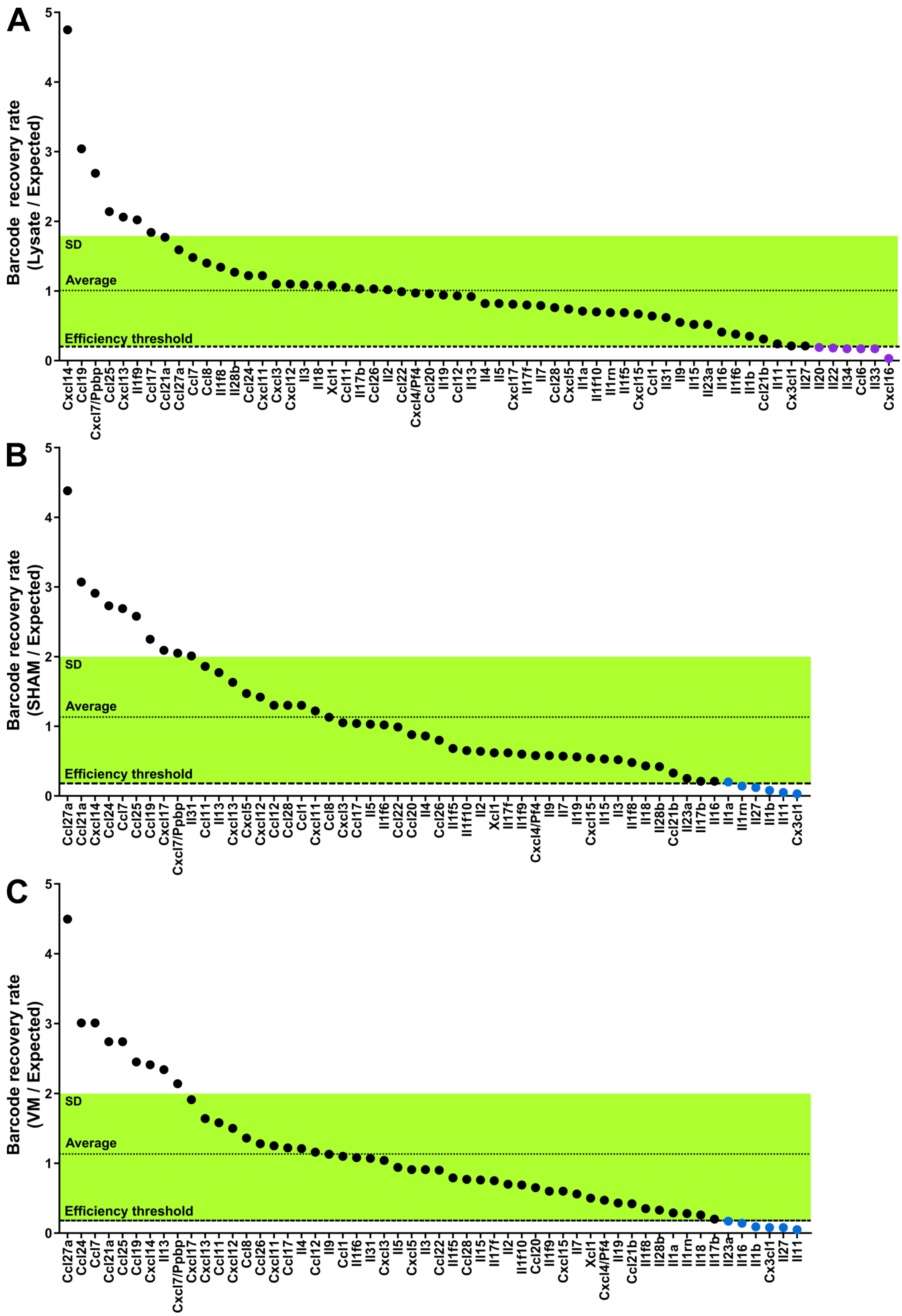

Figure S1: AAV9 Pool 60 barcode recovery rate is constant in the three biological sources. Relative barcode recovery of the Pool 60 in the initial lysate (A) and after FunSel in sham (B), and CVB3-infected hearts (C) at 14 dpi compared to the expected recovery (1/60). Efficiency threshold set at 0.20. Factors with a recovery rate below 0.20 in the lysate were discarded from further analysis as not sufficiently represented in the pool (violet dots). Factors with an enrichment below 0.20 either in sham or VM cardiac tissue were considered not efficiently overexpressed (blue dots). The green area represents 1 SD range from the average recovery rate.

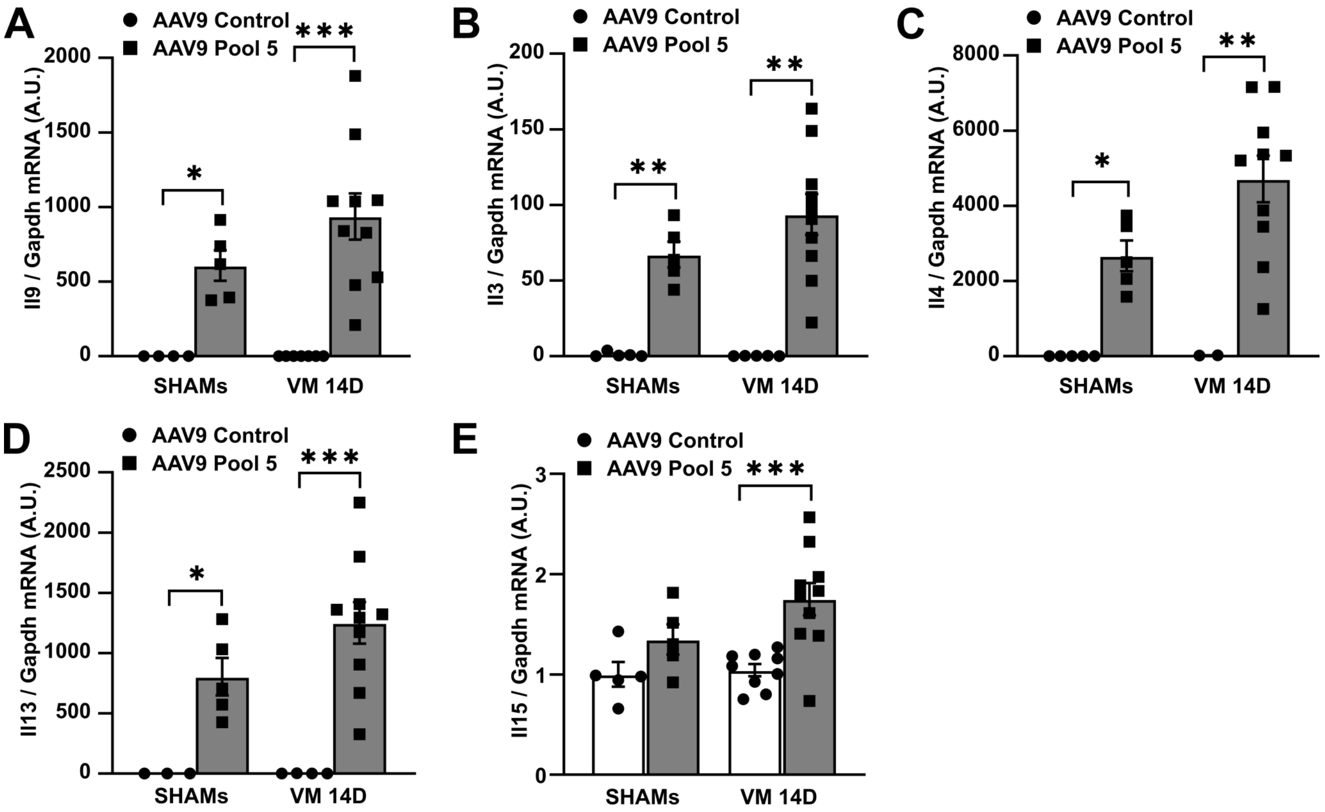

Figure S2: Efficient overexpression of IL9 plus IL3, IL4, IL13, IL15 (Pool 5) after acute VM. (A-E) Relative *Il3* (A), *Il4* (B), *Il9* (C), *Il13* (D), *Il15* (E) mRNA expression levels at 14 days post-infection in Pool 5-overexpressing hearts and controls. Significance assessed by two-way ANOVA followed by Tukey's test with *, p ≤ 0.05; **, p ≤ 0.01; ***, p ≤ 0.001.

Parameter	SHAMs AAV9 Control (n=6)	VM 35D AAV9 Control (n=17)	SHAMs AAV9 Pool5 (n=6)	VM 35D AAV9 Pool5 (n=17)
	Mean ± SEM	Mean ± SEM	Mean ± SEM	Mean ± SEM
Ejection fraction [%]	57 ± 3	37 ± 2***	55 ± 2	49 ± 2$^{\#\#\#}$
Cardiac output [ml/min]	23 ± 2	18 ± 1*	20 ± 1	21 ± 1$^{\#}$
End-distolic volume (EDV) [µl]	67 ± 6	83 ± 2*	66 ± 3	72 ± 3$^{\#}$
End-systolic volume (ESV) [µl]	29 ± 4	52 ± 3***	30 ± 2	37 ± 3$^{\#\#\#}$
Anterior wall in diastole (AWd) [µm]	718 ± 31	664 ± 26	748 ± 29	691 ± 22
Anterior wall in systole (AWs) [µm]	1003 ± 57	808 ± 31*	1103 ± 58	913 ± 35*
Left ventricular inner diameter in diastole (LVIDd) [µm]	3807 ± 85	4155 ± 54*	3722 ± 91	3978 ± 58
Left ventricular inner diameter in systole (LVIDs) [µm]	2291 ± 154	3552 ± 65***	2725 ± 79	3164 ± 99$^{*\#\#}$
Posterior wall in diastole (PWd) [µm]	697 ± 29	781 ± 29	778 ± 30	728 ± 27
Posterior wall in systole (PWs) [µm]	888 ± 31	894 ± 36	944 ± 34	894 ± 35
Heart rate [bpm]	533 ± 15	546 ± 7	516 ± 19	548 ± 11

Table S2. Echocardiographic analysis of Pool5-overexpressing sham and CVB3-infected mice and corresponding controls at 35 days post-infection. Significance was assessed by two-way ANOVA followed by Tukey's test with *, p ≤ 0.05; **, p ≤ 0.01; ***, p ≤ 0.001 versus corresponding SHAM group and #, p ≤ 0.05; ##, p ≤ 0.01; ###, p ≤ 0.001 versus corresponding AAV9 control group. All values are expressed as mean ± SEM.

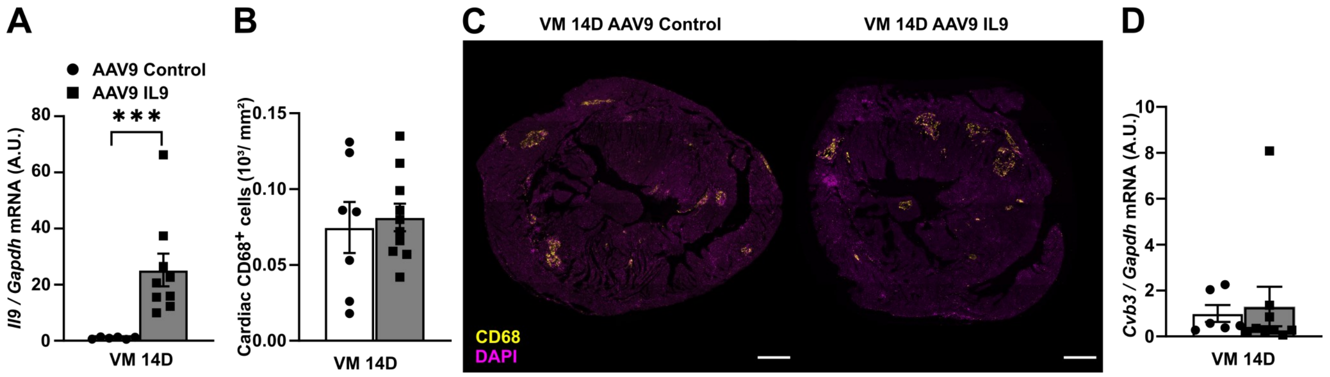

Figure S3: AAV9-IL9 reduces cardiac inflammatory response in acute VM

(A) Relative *Il9* expression levels at 14 days post-infection in IL9-overexpressing hearts and controls. Significance assessed by Mann-Whitney *U* test with ***, p ≤ 0.001. (B-C) CD68 staining (monocytes/ macrophages) in infected IL9-overexpressing and infected control hearts at 14 days post-infection. Scale bar = 500 µm. Significance assessed by Student's T-test. (D) relative *Cvb3* viral genome expression levels at 14 days post-infection in IL9-overexpressing hearts and controls. Significance assessed by Mann-Whitney *U* test. All values are expressed as mean ± SEM.

**Parameter**	**SHAMs AAV9 Control (n=4)**	**VM 35D AAV9 Control (n=8)**	**SHAMs AAV9 IL9 (n=4)**	**VM 35D AAV9 IL9 (n=8)**
	**Mean ± SEM**	**Mean ± SEM**	**Mean ± SEM**	**Mean ± SEM**
**Ejection fraction [%]**	41 ± 4	38 ± 2	37 ± 1	35 ± 2
**Cardiac output [ml/min]**	17 ± 2	17 ± 1	16 ± 1	14 ± 1
**End-distolic volume (EDV) [µl]**	84 ± 5	85 ± 3	90 ± 5	88 ± 4
**End-systolic volume (ESV) [µl]**	50 ± 5	53 ± 3	57 ± 4	57 ± 3
**Anterior wall in diastole (AWd) [µm]**	773 ± 50	697 ± 31	703 ± 29	763 ± 25
**Anterior wall in systole (AWs) [µm]**	1048 ± 47	905 ± 30	933 ± 49	1001 ± 38
**Left ventricular inner diameter in diastole (LVIDd) [µm]**	4098 ± 118	4278 ± 83	4275 ± 79	4301 ± 51
**Left ventricular inner diameter in systole (LVIDs) [µm]**	3235 ± 160	3614 ± 77	3594 ± 131	3618 ± 83
**Posterior wall in diastole (PWd) [µm]**	827 ± 58	691 ± 41	734 ± 40	726 ± 25
**Posterior wall in systole (PWs) [µm]**	1120 ± 58	851 ± 64	915 ± 70	908 ± 37
**Heart rate [bpm]**	484 ± 26	443 ± 22	508 ± 28	438 ± 23

Table S3: Echocardiographic analysis of IL9-overexpressing sham and CVB3-infected mice and corresponding controls at 35 days post-infection. Significance was assessed by two-way ANOVA, followed by Tukey's test. All values are expressed as mean ± SEM.
